# Prevalence of high-risk HPV genotypes, categorised by their quadrivalent and nine-valent HPV vaccination coverage, and the genotype association with high-grade lesions

**DOI:** 10.1186/s12885-018-4033-2

**Published:** 2018-01-30

**Authors:** María Paz-Zulueta, Ledicia Álvarez-Paredes, Juan Carlos Rodríguez Díaz, Paula Parás-Bravo, Ma. Encarnación Andrada Becerra, José María Rodríguez Ingelmo, María Montserrat Ruiz García, Joaquín Portilla, Miguel Santibañez

**Affiliations:** 1 Faculty of Nursing, University of Cantabria, Avda Valdecilla s/n. C.P.: 39008, Cantabria, Spain; 2 Department of Microbiology and Parasitology, University Hospital of Burgos, C/ Islas Baleares, 3 - C.P.: 09006, Burgos, Spain; 3 Department of Microbiology, University General Hospital of Alicante. Pintor Baeza, 11- C.P.: 03010, Alicante, Spain; 4 Department of Pathological Anatomy, University General Hospital of Elche. Camí de l’Almazara, 11 – C.P.: 03203, Alicante, Spain; 5 Department of Gynecology, University General Hospital of Elche. Camí de l’Almazara, 11 – C.P.: 03203, Alicante, Spain; 6 Department of Microbiology, University General Hospital of Elche. Camí de l’Almazara, 11 – C.P.: 03203, Alicante, Spain; 7 Department of Infectious Diseases, University Hospital of Alicante. Pintor Baeza, 11- C.P.: 03010, Alicante, Spain

## Abstract

**Background:**

The new nine-valent vaccine against human papillomavirus (HPV) includes the four HPV genotypes (6, 11, 16, and 18) that are targeted by the older quadrivalent HPV vaccine, plus five additional oncogenic types (31, 33, 45, 52, and 58) remain significantly associated with high grade lesions. We aimed to determine the prevalence of high-risk HPV genotypes in unvaccinated subjects and the association of these genotypes with the incidence of high-grade lesions. We also assessed which, if either, of these two HPV vaccines could have prevented these cases.

**Methods:**

This cross-sectional study, conducted from 4 January 2010 to 30 December 2011, was composed of 595 women attending the Hospital General Universitario de Elche (Spain) gynaecology department who were positively screened for opportunistic cervical cancer by pap smears and HPV detection during a routine gynaecological health check. The pap smear results were classified using the Bethesda system. HPV genotyping was performed with the Linear Array HPV genotyping test, and viruses were classified by the International Agency for Research on Cancer assessment of HPV carcinogenicity. Odds ratios (ORs) with their 95% confidence intervals (95% CI) were estimated by logistic regression, adjusting for age and immigrant status. The prevented fraction among those exposed (PFe-adjusted) was determined as a measure of impact.

**Results:**

At least one of the additional five high-risk HPV genotypes present in the nine-valent HPV vaccine was detected in 20.5% of subjects. After excluding women with genotype 16 and/or 18 co-infection, high-risk genotypes (31, 33, 45, 52, and 58) were associated with a higher risk of intraepithelial lesion or malignancy: adjusted OR = 3.51 (95% CI, 1.29–9.56), PFe-adjusted = 0.72 (95% CI, 0.22–0.90). Genotypes that are still non-vaccine-targeted were detected in 17.98% of the women, but these were not significantly associated with high-grade lesions.

**Conclusion:**

The greater protection of the nine-valent HPV vaccine is likely to have a positive impact because, in the absence of genotype 16 or 18 infection, these five genotypes on their own remained significantly associated with high-grade lesions.

**Electronic supplementary material:**

The online version of this article (10.1186/s12885-018-4033-2) contains supplementary material, which is available to authorized users.

## Background

Human papillomavirus (HPV) infection is the most common sexually transmitted infection in the United States and Europe [1]. Persistent HPV infection with a high-risk oncogenic genotype, as well as co-infection with high-risk genotypes, contributes to neoplastic progression [2–4]. Cervical cancer (CC) and other HPV-related cancers represent a major global public health problem. Cervical cancer is the third most common cancer among women worldwide [5]. It has been found that according to the severity of cervical lesions, the overall prevalence of HPV increases from 12% in women with normal cytology to 89% in women with CC [6]. The most frequent viral types are HPV 16, 18, 31, 33, 35, 45, 52 and 58. However, there are differences of specific type prevalences according to the region analysed. HPV 16 and 18 are the two most prevalent types worldwide [7]. HPV genotypes 16 and 18 cause 70% of cervical cancer cases as well as an even higher proportion of other cancers associated with HPV infection, such as cancer of the vulva, vagina, penis, anus, and oropharynx [8–10].

Two preventive strategies are used in combination to prevent cervical cancer: vaccination against HPV (primary prevention) and cervical screening programmes (secondary prevention). Cervical smears allow the detection of pre-cancerous lesions and cancer. Although it is a very effective method, its implementation only helps to prevent cervical cancer [11, 12]; there are no screening tests for other types of HPV-related cancer. In Spain, cervical screening is available but not mandatory. Cervical screening is performed opportunistically, its success depends on participant uptake.

In Europa, there are currently three vaccines authorised against HPV. The bivalent HPV vaccine Cervarix® provides protection against HPV 16 and 18 (high-risk oncogenic genotypes); the quadrivalent HPV vaccine Gardasil® provides protection against HPV 6 and 11 (low-risk oncogenic genotypes related with the appearance of 90% of genital warts) [13] together with genotypes 16 and 18. Nowadays in Spain, we use bivalent HPV vaccine Cervarix® and quadrivalent HPV vaccine Gardasil®. Depending on the region, one or other vaccine is included in the childhood immunization schedule. The last approved nine-valent HPV vaccine Gardasil9® provides protection against HPV 6, 11, 16, 18, 31, 33, 45, 52, and 58 [14]. This new vaccine extends protection to a further five oncogenic types (HPV 31, 33, 45, 52, and 58), in addition to the four types included in the original Gardasil®. Thus, the nine-valent HPV vaccine is estimated to potentially prevent close to 90% of cancers of the cervix, vulva, vagina, and anus, as well as 80% of pre-cancerous lesions [15]. Scientific evidence regarding the safety profile [16], immunogenicity [17, 18], and safety and efficacy [19–22] of these vaccines is well established.

The aims of our study were to determine the prevalence of high-risk HPV genotypes and analyse how effective the protection offered by the quadrivalent HPV vaccine and the new nine-valent HPV vaccine would be in this population as well as to investigate the association with and potential impact of these genotypes on the incidence of high-grade lesions.

## Methods

### Type of study

This was a cross-sectional study of 595 women.

### Population

During the study period (January 4,2010 to December 30,2011), women who came to the gynecology department of the Hospital General Universitario de Elche (Spain) for a routine gynecological check-up were treated according to the cervical cancer screening protocol: a combined Pap smear test and HPV screening. For our study, we included 595 consecutive women in whom the linear combined HPV genotyping test had detected HPV infection in a cervical smear.

### Data sources

We obtained the data for each patient from computerized hospital records of gynaecology and microbiology services. When necessary, this information was completed with data from paper medical records and the histopathology department Cliniviewer.

### Variables

The primary variables included date of birth, country of birth, patient’s VPH vaccine status, Pap smear result, and determination of HPV genotype by polymerase chain reaction (PCR) assay.

The results of the pap smears were classified according to the Bethesda 2001 histological classification [23]. Positive pap smears were defined as those that presented abnormal epithelial cells. The result was classified as: atypical squamous cells of undetermined significance or atypical glandular cells (ASC-US/AGC), low grade squamous intraepithelial lesion (LSIL), high grade squamous intraepithelial lesion (HSIL), squamous cell carcinoma, adenocarcinoma in situ, or adenocarcinoma.

HPV genotyping was performed using the Linear Array HPV genotyping test (Roche Diagnostics), a qualitative in vitro assay that utilizes the amplification of target DNA by PCR and nucleic acid hybridization and detects 37 HPV genotypes: 6, 11, 16, 18, 26, 31, 33, 35, 39, 40, 42, 45, 51, 52, 53, 54, 55, 56, 58, 59, 61, 62, 64, 66, 67, 68, 69, 70, 71, 72, 73 (MM9), 81, 82 (MM4), 83 (MM7), 84 (MM8), IS39, and CP6108. HPV types were classified according to the World Health Organization (WHO) International Agency for Research on Cancer (IARC) Monographs Working Group assessment of the carcinogenicity of different HPV types [24–26]: 13 high-risk (16, 18, 31, 33, 35, 39, 45, 51, 52, 56, 58, 59, and 68) and 5 likely high-risk (53, 66, 70, 73 MM9, and 82 MM4). The finding of two or more high-risk genotypes in the same patient was defined as co-infection [27].

Based on the protection that would have been provided by the quadrivalent or new nine-valent HPV vaccines, the high-risk genotypes were classified into [28]:

- vaccine-targeted high-risk HPV genotypes (16 and 18);
- quadrivalent HPV-targeted genotypes (6, 11, 16, and 18);
- the additional five high-risk HPV genotypes present in the nine-valent HPV vaccine (31, 33, 45, 52, and 58);
- high-risk HPV types other than 16 and 18 (31, 33, 35, 39, 45, 51, 52, 56, 58, 59, and 68); and
- other non-vaccine targeted high-risk types (35, 39, 51, 56, 59, and 68).

### Statistical analysis

For the categorical and discrete variables, proportions were estimated using the Pearson Chi-squared test to make comparisons and using Fisher’s exact test when necessary. Continuous variables were expressed as the mean and standard deviation (SD); a Student’s *t*-test was used for comparisons following normality testing using a Shapiro-Wilk test.

Cytological results were classified as being a “Non-high-grade lesion” (smear negative for intraepithelial lesion, ASC-US/AGC, or LSIL) or a “High-grade lesion” (HSIL, squamous cell carcinoma, adenocarcinoma in situ, or adenocarcinoma), and this classification was treated as a dependent variable in the logistic regression models.

As a measure of association, the crude odds ratios (ORc) as well as the odds ratios adjusted (ORa) for age (continuous variable) and immigrant status were estimated by unconditional logistic regression, together with their 95% confidence intervals (95% CI).

As a measure of impact, the adjusted attributable fraction in the total exposed (AFe-adjusted) was calculated using the formula [(ORa – 1)/ORa] together with its 95% CI using the formula [((lower limit 95% CI of the ORa – 1)/lower limit 95% CI of the ORa) to ((upper limit 95%CI of the ORa − 1)/upper limit 95% CI of the ORa)] when the ORa was statistically significant. The alpha error was set at 0.05, and all *p* were two-sided. All statistical analyses were performed using IBM SPSS Statistics V22.0.

## Results

Of the total participants, 73.11% (95% CI, 69.43–76.76) of women presented with at least one high-risk HPV genotype. High-risk HPV genotypes not targeted by the quadrivalent HPV vaccine were found in 38.49% of women (95% CI, 34.49–42.48%). At least one of the additional five high-risk HPV genotypes present in the nine-valent HPV vaccine (31, 33, 45, 52, or 58) was found in 20.50% of women (95% CI, 17.18–23.83%) (Table 1).

**Table 1:** High-risk HPV genotypes, vaccine-targeted high-risk HPV genotypes, and single type infections and multiple type infections

	Total				Single HPV type				Multiple HPV types^a^			
	*n* = 595	%	95% CI		*n* = 595	%	95% CI		*n* = 595	%	95% CI	
HPV genotypes												
Vaccine-targeted high-risk HPV genotypes												
16	180	30.08	26.32	33.85	65	10.92	8.33	13.52	115	19.33	16.07	22.59
18	32	5.38	3.48	7.28	6	1.01	0.12	1.90	26	4.37	2.64	6.10
Additional five high-risk HPV genotypes present in the nine-valent HPV vaccine												
31	56	9.41	6.98	11.84	19	3.19	1.70	4.69	37	6.22	4.19	8.24
33	27	4.71	2.92	6.49	6	1.01	0.12	1.90	21	3.53	1.96	5.10
45	18	3.03	1.57	4.49	4	0.67	0.18	1.71	14	2.35	1.05	3.66
52	85	14.29	11.39	17.18	8	1.35	0.34	2.35	77	12.94	10.16	15.72
58	37	6.22	4.19	8.24	4	0.67	0.18	1.71	33	5.55	3.62	7.47
Quadrivalent HPV and nine-valent HPV non-vaccine-targeted high-risk HPV genotypes												
35	17	2.86	1.43	4.28	3	0.50	0.10	1.47	14	2.35	1.05	3.66
39	46	7.73	5.50	9.96	9	1.51	0.45	2.58	37	6.22	4.19	8.24
51	69	11.60	8.94	14.25	14	2.35	1.05	3.66	55	9.24	6.83	11.66
56	55	9.24	6.83	11.66	2	0.34	0.04	1.21	53	8.91	6.54	11.28
59	28	4.71	2.92	6.49	6	1.01	0.12	1.90	22	3.70	2.10	5.30
68	13	2.19	0.93	3.44	0	0.00	0.00	0.62	13	2.19	0.93	3.44

^a^Women infected with multiple human papillomavirus (HPV) types: high-risk, likely high-risk HPV or low-risk genotypes [11]

High-risk HPV genotypes not targeted by either the quadrivalent HPV or the nine-valent HPV vaccine (35, 39, 51, 56, 59, or 68) were found in 17.98% of women (95% CI, 14.81–21.15%) (Table 1).

The mean age of the women included in this study was 34.34 years [SD: 10.70]; 89.75% were Spanish. Of the participants, 41.36% had at least one smear with morphological changes (ASC-US, LSIL, HSIL, squamous cell carcinoma, adenocarcinoma in situ, or adenocarcinoma). The prevalence of ASC-US was 8.14% (95% CI, 5.85–10.43), that of LSIL was 19.49% (95% CI, 16.21–22.77), and that of HSIL was 11.53% (95% CI, 8.86–14.19). The prevalence of cancerous lesions (squamous cell carcinoma, adenocarcinoma in situ, or adenocarcinoma) was 2.20% (95% CI, 0.93–3.47) (Additional file 1).

A single low-risk genotype (6, 11, 42, 43 or 44) was detected in 33.61% of the women (95% CI, 29.73–37.49), and a single likely high-risk genotype was detected in 25.88% (95% CI, 22.28–29.49). A single high-risk genotype was detected in 45.04% of participants (95% CI, 40.96–49.12), and two high-risk genotypes were detected in the 20.50% (Additional file 1). The most common multiple infections were by genotype 16 (19.33%), 52 (12.94%), or 51(9.24%) (Table 1). Genotype 16 was the most commonly detected HPV genotype, found in 30.08% of study participants (95% CI, 26.32–33.85). Genotypes 16 and/or 18 were detected in 35.46% of the women (Table 2). When we analysed the percentages of infection prevalence for the additional five high-risk HPV genotypes present in the nine-valent HPV vaccine, we found that 14.29% of women were infected by genotype 52, 9.41% by genotype 31, 6.22% by genotype 58, 4.71% by genotype 33, and 3.03% by genotype 45 (Table 1). Regarding the percentages of infection by high-risk HPV genotypes that are not targeted by either the quadrivalent or nine-valent HPV vaccine, we found that 11.60% of subjects were infected by genotype 51, 9.24% by genotype 56, 7.73% by genotype 39, 4.71% by genotype 59, 2.86% by genotype 35, and 2.19% by genotype 68 (Table 2).

**Table 2:** Characteristics of the studied population, distribution of HPV genotypes, and histological classification of cervical smears

	Total			
	*n* = 595	%	95% CI	
Age (years): Mean [SD]	34.34	[10.70]	33.48	35.20
Country of origin				
Spain	534	89.75	87.23	92.27
Other	61	10.25	7.73	12.77
Number low-risk oncogenic HPV^a^				
1 genotype	200	33.61	29.73	37.49
2 genotypes	63	10.59	8.03	13.15
3 genotypes	15	2.52	1.18	3.87
4 genotypes	6	1.01	0.12	1.90
Number likely high-risk oncogenic HPV^a^				
1 genotype	154	25.88	22.28	29.49
2 genotypes	12	2.02	0.80	3.23
3 genotypes	1	0.17	0.00	0.93
Number high-risk oncogenic HPV^a^				
1 genotype	268	45.04	40.96	49.12
2 genotypes	122	20.50	17.18	23.83
3 genotypes	30	5.04	3.20	6.88
4 genotypes	14	2.35	1.05	3.66
5 genotypes	1	0.17	0.00	0.93
Results cervical smear^b^				
NILM^c^	272	46.10	42.00	50.21
Inflammatory	70	11.86	9.17	14.56
Warts	1	0.17	0.00	0.94
ASC-US/ASC-H	48	8.14	5.85	10.43
LSIL	115	19.49	16.21	22.77
HSIL	68	11.53	8.86	14.19
Carcinoma/Adenocarcinoma	13	2.20	0.93	3.47
Result not available	3	0.51	0.11	1.48
Missing	5			

^a^Classification of human papillomavirus (HPV) types according to the World Health Organization International Agency for Research on Cancer (IARC) Monographs Working Group assessment of the carcinogenicity of different HPV types [25–27]

^b^Histological classification Bethesda 2001 System [24]

^c^Negative for intraepithelial lesion or malignancy

Table 3 shows the crude and adjusted measures of association (OR) and impact (AFe) of the risk of high-grade lesions, in relation to the HPV genotype and its potential protection by the quadrivalent and nine-valent HPV vaccines. After adjusting for age and immigrant status, the presence of HPV genotype 16 or 18 individually increased the possibility of having a high-grade lesion by 10.40-fold. The concomitant presence of both of these genotypes increased the risk of lesion as follows: adjusted OR = 63.58; linear *p* trend < 0.001; AFe = 0.98 (95% CI, 0.88–1.00). The presence of a high-risk genotype not covered by the quadrivalent HPV vaccine increased the risk of a high-grade lesion significantly, although to a lesser extent than the presence of genotype 16 or 18: adjusted OR = 2.71 (95% CI, 1.05–7.01).

**Table 3:** Prevalence of high-risk human papillomavirus genotypes according to the vaccine-targeted high-risk human papillomavirus genotypes

		Tota**l**			
		n = 595	%	95%CI	
Regarding HPV genotype					
No high-risk^a^		160	26.89	23.24	30.54
At least 1 high-risk		435	73.11	69.46	76.76
According to the vaccine-targeted high-risk HPV genotypes					
No high-risk^a^		160	26.89	23.24	30.54
Quadrivalent HPV non-vaccine-targeted high-risk HPV genotypes^b^		229	38.49	34.49	42.48
	Quadrivalent HPV and nine-valent HPV non-vaccine-targeted high-risk HPV genotypes^c^	107	17.98	14.81	21.15
	Additional five high-risk HPV genotypes present in the nine-valent HPV vaccine^d^	122	20.50	17.18	23.83
Vaccine-targeted high-risk HPV genotypes (16 or 18)		201	33.78	29.90	37.67
Vaccine-targeted high-risk HPV genotypes (16 and 18)		5	0.84	0.27	1.95

^a^No high-risk. Classification human papillomavirus (HPV) types according to the World Health Organization International Agency for Research on Cancer (IARC) Monographs Working Group assessment of the carcinogenicity of different HPV types [25–27]

^b^At least 1 of the quadrivalent HPV non-vaccine-targeted high-risk HPV genotypes (31 or 33 or 35 or 39 or 45 or 51 or 52 or 56 or 58 or 59 or 68)

^c^At least 1 of the quadrivalent HPV and nine-valent HPV non-vaccine-targeted high-risk HPV genotypes (35 or 39 or 51 or 56 or 59 or 68)

^d^At least 1 of the additional five high-risk HPV genotypes present in the nine-valent HPV vaccine (31 or 33 or 45 or 52 or 58)

By sub-dividing these genotypes based on the ability of the new nine-valent HPV vaccine to protect against them, we found that although the presence of high-risk genotypes not covered by the new nine-valent HPV vaccine was not associated with a significant increase in the risk of lesions (adjusted OR = 1.76; 95% CI, 0.54–1.70), the presence of a high-risk genotype not covered by the quadrivalent HPV vaccine but covered by the new nine-valent HPV vaccine was associated with a significant increase in this risk (adjusted OR = 3.51, 95% CI, 1.29–9.56; adjusted AFe = 0.72, 95% CI, 0.22–0.90) (Table 4).

**Table 4:** Associations and impact between the risk intraepithelial lesion/malignancy and the vaccine-targeted high-risk HPV genotypes

		No lesion	Intraepithelial lesion or malignancy^a^									
		*n* = 509	*n* = 81	ORc	95%CI		ORa	95%CI		AFe-a	95%CI	
Regarding HPV genotype												
No high-risk^b^		154	6	1	–		1	–				
At least 1 high-risk		355	75	5.42	2.31	12.72	6.01	2.53	14.29	0.83	0.60	0.93
According to the vaccine-targeted high-risk HPV genotypes												
No high-risk^b^		154	6	1	–		1	–				
Quadrivalent HPV non-vaccine-targeted high-risk HPV genotypes^c^		208	20	2.47	0.97	6.29	2.71	1.05	7.01	0.63	0.04	0.86
	Quadrivalent HPV and nine-valent HPV non-vaccine-targeted high-risk HPV genotypes^d^	101	6	1.53	0.48	4.86	1.76	0.54	5.70	0.43	–	–
	Additional five high-risk HPV genotypes present in the nine-valent HPV vaccine^e^	107	14	3.36	1.25	9.02	3.51	1.29	9.56	0.72	0.22	0.90
Vaccine-targeted high-risk HPV genotypes (16 or 18)		145	52	9.21	3.84	22.08	10.40	4.25	25.40	0.90	0.76	0.96
Vaccine-targeted high-risk HPV genotypes (16 and 18)		2	3	38.50	5.39	275.06	63.58	8.62	468.70	0.98	0.88	1.00
				*< 0.001*			*< 0.001*					

^a^Intraepithelial lesion or malignancy: high-grade squamous intraepithelial lesion or worse

Odds ratio and 95% confidence intervals. ORc denotes “crude OR”; ORa denotes “adjusted OR” OR adding to the basic model: age and immigrant status

AFe-a: Attributable fraction among the exposed, adjusted for age and immigrant status

^b^No high-risk. Classification of human papillomavirus (HPV) types according to the World Health Organization International Agency for Research on Cancer (IARC) Monographs Working Group assessment of the carcinogenicity of different HPV types [25–27]

^c^At least 1 of the quadrivalent HPV non-vaccine-targeted high-risk HPV genotypes (31 or 33 or 35 or 39 or 45 or 51 or 52 or 56 or 58 or 59 or 68)

^d^At least 1 of the quadrivalent HPV and nine-valent HPV non-vaccine-targeted high-risk HPV genotypes (35 or 39 or 51 or 56 or 59 or 68)

^e^At least 1 of the additional five high-risk HPV genotypes present in the nine-valent HPV vaccine (31 or 33 or 45 or 52 or 58)

## Discussion

Our results show that in the absence of infection with HPV genotypes 16 or 18, the greater level of protection provided by the new nine-valent HPV vaccine compared with that provided by the quadrivalent HPV vaccine is likely to have a measurable impact. The extra five HPV genotypes remained significantly associated with high grade lesions.

Our results show a high prevalence of infection by a single high-risk oncogenic HPV genotype (45.04%) [29–32]. The percentage of women who presented a morphological change in their pap smear (positive screening) was also high (41.36%) [33, 34]. This could be due to the characteristics of our sample, since our study was not conducted on the general population but rather it specifically included women with a confirmed HPV infection. Similarly, with respect to the prevalence of genotypes 16 and 18 in our sample, we found higher prevalences (30.08% and 5.38%, respectively) than those described in previous population-based studies [29, 32]. Our findings are closer to those reported by Garcia Espinosa et al. [35], who showed that the most common HPV genotypes found in all specimens were genotypes 16 (26.0%), 31 (10.7%), and 58 (8.0%). In their study, genotype 18 was detected in only 5.0% of women.

Here, the prevalence of genotype 52 was higher than that found by other authors in national studies [36, 37]. Our results also do not coincide with the prevalence reported by the 2016 ICO-WHO report [38], which detected genotype 52 in 5.4% of HSIL and in 2.4% of cancerous lesions. This difference could be due to the high population of immigrants from East Africa that plays an important role in our health area; a very high prevalence of HPV 52 infection has been reported for this geographic area [39]. In contrast, the data published by Castellsagué et al. and Delgado et al. [30, 40] regarding the prevalence of HPV 52 are similar to those obtained in our study. Castellsagué et al. found that after HPV 16, genotype 52 was the most prevalent, and Delgado et al. reported the prevalence for genotype 52 as 12%.

The most common high-risk type detected in this study was HPV16 which is consistent with published studies and that vaccination will likely have a significant impact [20–22, 41]. Furthermore, HPV genotype 16 or 18 cause 70% of cases of cervical cancer and an even higher proportion of cancers of the vulva, vagina, penis, anus, and oropharynx [8–10]. Thus, current HPV vaccines (Cervarix® and Gardasil®) prevent approximately 70% of cervical cancers through protection against genotypes 16 and 18.

Partial cross-protection against non-vaccine targeted HPV types has been reported for both licensed vaccines, although the clinical significance of this finding remains uncertain [42]. In the FUTURE I / II and PATRICIA studies, conducted in young women, the bivalent vaccine showed significant efficacy against HPV31, 33, 45 and 52, and the tetravalent vaccine against HPV31. The bivalent vaccine showed significant efficacy against CIN2 + related to HPV31 and 33 (excluding co-infection with HPV16 and / or 18) and related to HPV45 (included co-infection with HPV16 and / or 18) [43, 44]. The possible cross-protection of the new nine-valence vaccine is still unknown. It would be necessary to evaluate whether the nine-valence vaccine also protects against other non-HPV oncogenic HPV types not included in this vaccine.

Concomitant infection by both genotypes 16 and 18 also suggests an interaction, with co-infection multiplicatively increasing the risk of high-grade lesions (adjusted OR = 63.58), with an adjusted AFe of 90% and 98% for isolated or joint infection, respectively. Thus, protection against infection with one of these genotypes (16 or 18) would prevent approximately 90% of high-grade lesions, while protection against both would prevent 98% of the high-grade lesions present in women co-infected with genotypes 16 and 18. These findings support the preventive impact of both the quadrivalent and the nine-valent HPV vaccine, as both cover genotypes 16 and 18 [19, 42, 45, 46].

The new nine-valent HPV virus-like particle includes the four HPV types (6, 11, 16 and 18) present in the quadrivalent HPV vaccine, plus five additional oncogenic types (31, 33, 45, 52, and 58) and potentially increases the overall prevention of cervical cancer from around 70% to around 90% [47]. Our study supports this increased efficacy. The greater level of protection by the new nine-valent HPV vaccine compared with that of the quadrivalent HPV vaccine is likely to have an observable impact, given our finding that these genotypes on their own (in the absence of infection by genotypes 16 or 18) remained significantly associated with high grade lesions, with an adjusted AFe of 72%. Thus, our results suggest that had the 121 patients who presented with at least one of these HPV genotypes (but not genotype 16 or 18) been vaccinated with the new nine-valent HPV vaccine, approximately 72% of the high-grade lesions present in these women would have been prevented. In other words, if all these 121 patients had been vaccinated, and the efficacy of the vaccination for these genotypes (31, 33, 45, 52, and 58) was 100%, approximately 87 high-grade lesions would have been prevented. Joura et al. showed an efficacy of 96.7% against high-grade lesions related with infection by HPV 31, 33, 45, 52, and 58 in their analysis of a cohort of 14,215 women aged 16 to 26 years [22]. Monsonego et al. showed that a high proportion of high-grade cervical lesions (60.6% of genotyping assay-positive CIN2+) were associated with HPV types 31, 33, 45, 52, or 58 [48].

In our study, 17.98% of women were infected with high-risk genotypes that are not covered by either the quadrivalent or the new nine-valent HPV vaccine. In the absence of infection with genotypes 16 or 18, infection with the remaining high-risk genotypes not covered by the new nine-valent HPV vaccine (genotypes 35, 39, 51, 56, 59, and 68) was associated with a non-statistically significant risk of high-grade lesions, with an AFe close to 40%.

When extrapolating our results, various aspects that could result in lower attributable fractions in the population must be considered. The effectiveness of the HPV vaccines is likely less than 100%, and therefore the protection level is not 100% in 100% of the women vaccinated. Furthermore, we cannot rule out the false sense of security reported by several authors [49, 50], which can lead to a higher prevalence of risky lifestyles or sexual habits, such as a lower use of condoms. Another limitation is the fact that the attribution of lesions to specific HPV types is complicated by the detection of multiple HPV infections. Therefore, the potential benefit of a HPV vaccine is not easily assessable.

On the other hand, another caveat is that cervical lesion was performed on exfoliated cervical cells instead of biopsy samples with laser dissection, so we did not have confirmation of high-grade lesions by histological study (biopsy). However, studies evaluating the category HSIL compared to biopsy as gold standard support a very high probability of an accurate diagnosis [47, 51].

In our study, the enrolled women had attended the gynaecology department of the Hospital General Universitario de Elche (eastern Spain) testing positive for HPV infection in the cervical smear performed during a routine gynaecological health check in an opportunistic cervical screening program. Since the population is limited to those attending opportunistic screening further selected on the basis of their HPV status and not primarily vaccine status, it prevents to make generalizations on potential vaccine impact in the general population. However, our results circumscribed to the study population, show the prevalence of high-risk HPV genotypes in unvaccinated subjects and the association of these genotypes with the presence of high-grade lesions, allowing to assess the potential impact of quadrivalent and nine-valent HPV vaccines by using epidemiological impact measures. One advantage of using adjusted ORs is that derived from these adjusted OR, it is possible to estimate as a measure of impact, the adjusted attributable fraction in the total exposed together with its 95%CI. We think that our methodology can complement the information provided with other methodologies such as the one followed by the recently published study by Capra et al. [52], and that it could be useful for future reviews or meta-analysis. The description of high-risk HPV epidemiology is a key feature for the design of strategies to prevent cervical cancer, and the results from this study can be used with this purpose in future reviews and meta-analysis, in order to estimate the potential impact of vaccination by either the quadrivalent or nine-valent vaccine relative to other studies with similar aims.

## Conclusion

HPV genotypes 16 and 18, especially in co-infection, are the two HPV genotypes that have most impact on the presence of high-grade lesions in our unvaccinated population attending an opportunistic screening in eastern Spain. Based on our associations and impact epidemiological results, in the absence of infection with HPV genotypes 16 or 18, the greater level of protection provided by the new nine-valent HPV vaccine (five genotypes) compared with that provided by the quadrivalent HPV vaccine is likely to have a measurable impact because these genotypes on their own remained significantly associated with high-grade lesions. In our population, there is a percentage of women (17.98%) who are infected with high-risk HPV genotypes that are not covered by any of the currently available vaccines (quadrivalent HPV or nine-valent HPV vaccines); however, as a whole, the impact of these HPV genotypes on the risk of high-grade lesions appears to be lower than the risk posed by other common HPV genotypes.

## Additional file

Additional file 1: Characteristics of the studied population, distribution of HPV genotypes, and histological classification of cervical smears. (DOCX 18 kb)

## Abbreviations

AFe: Adjusted attributable fraction in the exposures
AGC: Atypical glandular cells
ASC-US: Atypical squamous cells of undetermined significance
CI: Confidence intervals
HPV: Human papillomavirus
HSIL: High grade squamous intraepithelial lesion
IARC: World health organization international agency for research on cancer
LSIL: Low grade squamous intraepithelial lesion
OR: Odds ratios
ORa: Odds ratios adjusted
ORc: Crude odds ratios
PCR: Polymerase chain reaction
PFE: Prevented fraction among the exposed
SD: Standard deviation
