# Supplementary material for: Prevalence of high-risk HPV genotypes, categorised by their quadrivalent and nine-valent HPV vaccination coverage, and the genotype association with high-grade lesions
Source: BMC Cancer. 2018 Jan 30;18:112. doi: 10.1186/s12885-018-4033-2 (PMC5791190; doi:10.1186/s12885-018-4033-2)
Supplement: Additional file 1: — Characteristics of the studied population, distribution of HPV genotypes, and histological classification of cervical smears. (DOCX 18 kb) [file 12885_2018_4033_MOESM1_ESM.docx]

**Supplementary Table 1. Characteristics of the studied population, distribution of HPV genotypes, and histological classification of cervical smears.**

|  |  | **Total** |  |  |  |
| --- | --- | --- | --- | --- | --- |
|  |  | n=595 | % | 95% CI |  |
| **Age (years): Mean [SD]** |  | 34.34 | [10.70] | 33.48 | 35.20 |
| **Country of origin** |  |  |  |  |  |
|  | Spain | 534 | 89.75 | 87.23 | 92.27 |
|  | Other | 61 | 10.25 | 7.73 | 12.77 |
| **Results cervical smear^a^** |  |  |  |  |  |
|  | NILM^b^ | 272 | 46.10 | 42.00 | 50.21 |
|  | Inflammatory | 70 | 11.86 | 9.17 | 14.56 |
|  | Warts | 1 | 0.17 | 0.00 | 0.94 |
|  | ASC-US/AGC | 48 | 8.14 | 5.85 | 10.43 |
|  | LSIL | 115 | 19.49 | 16.21 | 22.77 |
|  | HSIL | 68 | 11.53 | 8.86 | 14.19 |
|  | Carcinoma/  Adenocarcinoma | 13 | 2.20 | 0.93 | 3.47 |
|  | Result not available | 3 | 0.51 | 0.11 | 1.48 |
|  | Missing | 5 |  |  |  |
| **Number low-risk oncogenic HPV^c^** |  |  |  |  |  |
|  | 1 genotype | 200 | 33.61 | 29.73 | 37.49 |
|  | 2 genotypes | 63 | 10.59 | 8.03 | 13.15 |
|  | 3 genotypes | 15 | 2.52 | 1.18 | 3.87 |
|  | 4 genotypes | 6 | 1.01 | 0.12 | 1.90 |
| **Number likely high-risk oncogenic HPV^c^** |  |  |  |  |  |
|  | 1 genotype | 154 | 25.88 | 22.28 | 29.49 |
|  | 2 genotypes | 12 | 2.02 | 0.80 | 3.23 |
|  | 3 genotypes | 1 | 0.17 | 0.00 | 0.93 |
| **Number high-risk oncogenic HPV^c^** |  |  |  |  |  |
|  | 1 genotype | 268 | 45.04 | 40.96 | 49.12 |
|  | 2 genotypes | 122 | 20.50 | 17.18 | 23.83 |
|  | 3 genotypes | 30 | 5.04 | 3.20 | 6.88 |
|  | 4 genotypes | 14 | 2.35 | 1.05 | 3.66 |
|  | 5 genotypes | 1 | 0.17 | 0.00 | 0.93 |

^a^Histological classification Bethesda 2001 System [24].

^b^Negative for intraepithelial lesion or malignancy.

^c^Classification of human papillomavirus (HPV) types according to the World Health Organization International Agency for Research on Cancer (IARC) Monographs Working Group assessment of the carcinogenicity of different HPV types [25-27].
